# Exploring barriers and enabling factors for surgical task sharing with physician assistants in Liberia: a qualitative pre-implementation study

**DOI:** 10.1136/bmjopen-2023-081363

**Published:** 2024-07-16

**Authors:** Markus Jan Werz, Alex J van Duinen, Theophilus C Hampaye, Ankie van den Broek, Håkon A Bolkan

**Affiliations:** 1 Amsterdam UMC, Amsterdam, Noord-Holland, The Netherlands; 2 Department of Public Health and Nursing, Norwegian University of Science and Technology, Trondheim, Trøndelag, Norway; 3 Clinic of Surgery, St. Olavs hospital, Trondheim University Hospital, Trondheim, Norway; 4 Liberia National Physician Assistant Association (LINPAA), Monrovia, Liberia; 5 Royal Tropical Institute, Amsterdam, The Netherlands

**Keywords:** PUBLIC HEALTH, Implementation Science, SURGERY

## Abstract

**Abstract:**

**Objectives:**

This study explores potential barriers and enabling factors that may influence the acceptance of implementation of a surgical task-sharing initiative targeting physician assistants (PAs) in Liberia.

**Design:**

A qualitative, pre-implementation study using semistructured interviews. Data was analysed in NVivo V.12 using deductive coding and the consolidated framework for implementation research as a guide.

**Setting:**

Liberia has few surgical providers and a poor surgical infrastructure resulting in a very low surgical volume. The research was conducted in the context of an already running surgical task-sharing programme for midwives.

**Participants:**

In 2019, a total of 30 key stakeholders in the field of surgery and the PAs training programme were interviewed.

**Results:**

The majority of the stakeholders supported the idea of training PAs in surgery. The high unemployment rate among PAs and the need for career advancement of this cadre were important enabling factors. Resistance against surgical task sharing for mid-level clinicians is multifaceted. The Ministry of Health (MOH) did not share a common vision. Opponents within the MOH believed budgetary constraints within the MOH and the lack of surgical infrastructure is a more pressing problem compared with the surgically trained human resources. Another important group of opponents are medical officers (MOs) and their professional bodies. Many of their negative beliefs around surgical task sharing reflect lessons to be drawn from the current surgical training programme for midwives.

**Conclusion:**

Prior to deciding on implementation of a surgical training programme for PAs, wider support is needed. If surgical task sharing with PAs is to be considered, the intervention should focus on adapting the ‘adaptable’ periphery of the intervention to broaden the support of the MOH, MOs and their professional bodies. Failing to obtain such support should make the implementors consider alternative strategies to strengthen surgical human resources in rural Liberia.

**Strengths and limitations of this study**

- Utilisation of the consolidated framework for implementation research adds a structured and theoretically informed approach to the study.
- Authors’ extensive experience with a task-sharing programme in neighbouring Sierra Leone enhances the credibility and depth of the study.
- A limitation is that important stakeholders such as patients and the community were not interviewed.
- Because of limited availability of medical specialists, it was difficult to compare the opinions of surgeons and gynaecologists.
- While the lead author (MJW) is an experienced medical doctor with expertise in global health and surgery, having a single researcher conduct the interviews might have introduced potential bias or subjectivity in the data collection process.

## Introduction

 Nearly one-third of the burden of human disease worldwide is amenable to surgery.^1^ Surgery is a cross-cutting intervention, at all ages, involved in every disease category. Currently, there is an increased global interest and effort on improving access to essential surgical care in low-income and middle-income countries. It is estimated that 5 billion people lack access to safe and timely surgery.^2^ Shortage of human resources and geographical maldistribution are two main factors contributing to the lack of available surgical and obstetric emergency services.^3 4^ Surgical task sharing is a strategy to increase access to surgical services by delegating tasks from surgical specialists to non-specialist medical officers (MOs) and to associate clinicians like physician assistants (PAs) or midwives.

Important benefits of surgical task sharing towards a cadre with fewer qualifications are the reduced training time, fewer employment costs and higher retention rates in rural areas. It is highly cost-effective and can increase accessibility to and availability of surgical care^5^ without compromising the quality and safety of care.^6 7^ The WHO supports the concept of surgical task sharing in countries which face a human resource crises within the field of surgery.^8 9^ Multiple studies from different African countries, comparing surgical outcomes of associate clinicians with MOs, found no significant differences in emergency maternal care or in general surgery.^510^,^12^

A recent a countrywide observational survey found a surgical volume of 462 operations per 100 000 population per year in Liberia,^13^ which is far below the recommended 5000 surgeries per 100 000 population per year set by the Lancet Commission on Global Surgery.^2^ Prior to developing an intervention to strengthen surgical human resources in Liberia, we aimed to assess barriers and enabling factors that may influence the implementation of a surgical task-sharing programme for PAs.

CapaCare, an organisation involved in training associate clinicians in surgery in Sierra Leone,^14^ was interested to explore the opportunity of extending its activities to the context of neighbouring Liberia. Therefore, outcomes of this study were used to guide their strategic direction.

## Methods

### Study setting

Liberia is a country in West Africa of 4.5 million inhabitants. A decade long civil war and the 2014–2016 Ebola epidemic resulted in a fragile healthcare system. The WHO estimates the need for at least 4.45 physicians, nurses and midwives per 1000 population. In 2015, when including the PAs, Liberia had 0.63 physicians, nurses and midwives per 1000 population (14% of recommended).^15^

Furthermore, MOs are unequally distributed with 61% working in Montserrado county, mostly in urban areas, and caring for one-third of the population.^16^ During the rainy season, large areas in the interior are practically inaccessible affecting health-seeking behaviour and possibilities for referral.

### Physician assistants

Since 1958 Liberia has implemented a programme for PAs. PAs work mostly independently, especially, at (rural) health posts, clinics and health centres using the basic concepts of primary healthcare. In places where no trained midwife is available, he or she could also provide basic obstetrical care. At the moment, Liberia has three PA training institutions (online supplemental material 1). In 2019, there were 1036 registered PAs, of which 532 were actively practising clinical medicine, suggesting many PAs are not practising or not being recorded as practising (49%). Of the group practising, 75% were working in the public and 25% in the private sector. From the group working in public sector, 80% is working in primary healthcare and the other 20% in the hospitals (source: PA Association). The exact number of PAs involved in surgery or independently performing surgeries is unknown but expected to be low.

### Surgical task sharing

In the literature, the terms ‘task shifting’ and ‘task sharing’ are used interchangeably. In this manuscript, we consciously choose the term ‘task sharing’ as this underlines a broader systemic approach and the necessary support from MOs to deliver safe and high-quality surgical care together with an eventually newly trained cadre.^17^

In 2018, Liberia had 286 registered surgical providers, including 67 medical specialists and 19 non-physicians. Areas with higher poverty had fewer specialists (0.7 per 100 000) compared with less impoverished areas (3.6 per 100 000). Non-specialist physicians (MOs) performed 58.3% of surgeries.^18^ A 6-month period during the training of MOs is dedicated to obtaining skills in emergency obstetrical surgeries and neonatal care.

Additionally, anaesthesia is mostly provided by anaesthetic nurses trained at Phebe Hospital.

In 2009, the Liberian Ministry of Health (MOH) participated in a conference on task sharing with associate clinicians in Addis Ababa, Ethiopia. This resulted in the development of a document in which the MOH supported the concept of task sharing, especially within the field of maternal and neonatal health.^19^ Maternal and Child health Advocacy International, an international non-governmental organisation from the UK, used this statement to justify the start of a surgical task-sharing programme training midwives, called clinical obstetricians (COs) to perform obstetric surgeries in Liberia.^20^ Within this training programme, the trainees started assisting senior doctors but progressed to independently manage obstetric surgeries. In April 2019, the WHO published an external evaluation of the Liberian COs’ programme concluding positively about the performance on patient outcomes and cost-effectiveness.^19^ The report also highlighted the challenge that key stakeholders, most importantly the Liberian Medical and Dental Council (LMDC), opposed the training of COs, who were of the opinion of not given the opportunity to voice their concerns against the new training initiative.

### Surgical task-sharing in neigbouring Sierra Leone

Sierra Leone and Liberia share similar healthcare challenges, including a high unmet need for surgery^13 21^ and weak healthcare systems. CapaCare, operating as both an international and national NGO, started a surgical training programme for associate clinicians in obstetric and general surgery in Sierra Leone in 2011. At the start, the training programme was designed for community health officers, a cadre comparable to PAs in Liberia, both working mainly in primary healthcare facilities. This programme involves 12 months of basic training in a main training facility followed by clinical rotations in partner hospitals. After completing rotations and examinations, graduates undergo a 1-year housemanship stage, split between tertiary hospitals in Freetown and district hospitals.^14^

### Study design and data collection

This qualitative study consists of semistructured key informant interviews (n=30) with key actors within the field of surgery and/or involved with the training of PAs in Liberia. It explores the participants’ views on surgical task sharing with special focus on the idea of implementing a ‘hypothetical’ surgical task-sharing training programme for PAs in the future. The semistructured interviews were guided by themes distilled through a combination of literature identified and discussion among the research team (online supplemental material 2). The general format of the semistructured interview guide was pretested with the assistant researcher to gauge understanding within the Liberian setting. The interviews were performed in English by a Dutch medical doctor specialised in global health and tropical medicine and with experience with a surgical task-sharing programme in Sierra Leone.^14^ The local assistant researcher joined to facilitate logistics and interpretation. Interviews were recorded and transcribed. Interviews lasted from 20 to 90 min depending on the input of the participants. Interviews were performed in the last quarter of 2019.

### Patients and public involvement statement

Patients or the public were not actively involved in the design, conduct, reporting or dissemination plans of our research.

### Sampling

**Figure 1:**
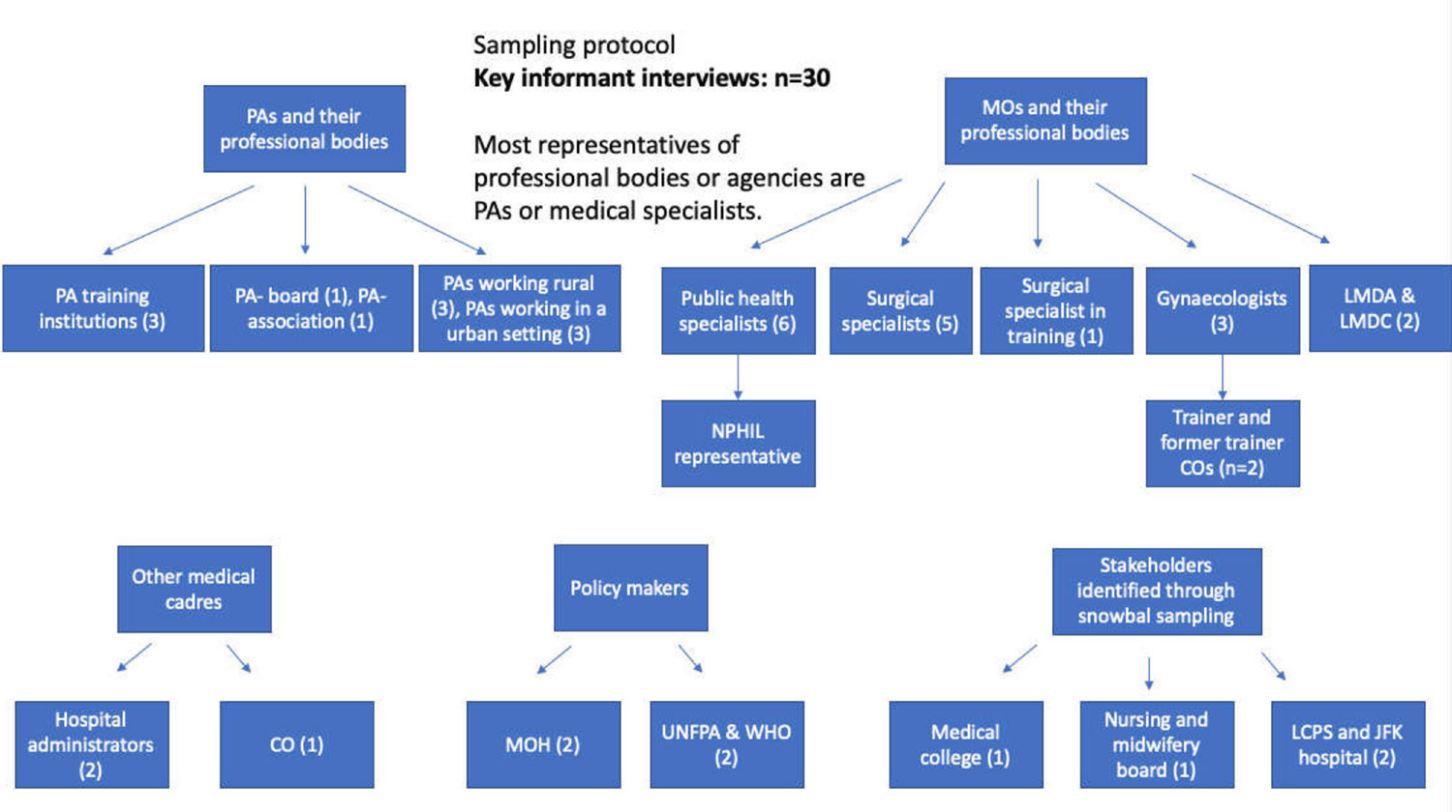
Purposive sampling protocol of the key stakeholders interviewed (n=30). COs, clinical obstetricians; JFK, John F. Kennedy Hospital; LCPS, Liberian College of Physicians and Surgeons; LMDA, Liberian Medical and Dental Association; LMDC, Liberian Medical and Dental Council; MOs, medical officers; MOH, Ministry of Health; NPHIL, National Public Health Institute of Liberia; PAs, physician assistants; UNFPA, United Nations Family Planning Agency.

Actors were identified through discussion within the research team and additionally through snowball sampling (asking all participants: ‘who are the most influential stakeholders in the field of surgery? And why?’). The qualitative sampling was purposive and is shown in figure 1. More stakeholders were identified but not interviewed as they were not expected to deliver new key insights, as shown in table 1. The study acknowledges that patients and the community are important stakeholders as well but were not included in the interviews. Surgical task sharing is already common in Liberia, involving non-specialist physicians, midwives and anaesthetic nurses. Consequently, in general, it is recognised as an accepted intervention by patients and the community.

**Table 1:** Stakeholders identified, description and interviewed (yes or no)

Stakeholder	Description	Interviewed
Ministry of Health (MOH): key representatives	Main government regulatory and decision-making body concerning (public) health affairs.	Yes
Physician assistants (PAs)	PAs working in an urban, rural, private and public sector were interviewed.	Yes
PA training institutions: key representatives	All three PA (public and private) training institutions were interviewed.	Yes
PA Association and PA Board	Official regulatory bodies of the PAs.	Yes
Liberian Medical and Dental Association: key representative	Association of MOs or medical doctors with a close link to the LMDC and with a large influence within the medical sector.	Yes
Liberian Medical and Dental Council: key representative	Regulatory body of MOs with a large influence within the medical sector.	Yes
Medical officers (MOs)	General practitioners or non-specialised MOs or doctors.	Yes
Surgeons and gynaecologists	MOs specialised in surgery or gynaecology. Senior authorities in the field of surgery and gynaecology in the country.	Yes
AM Dogliotti College of Medicine: key representative	The Liberian Medical College.	Yes
JFK Hospital: key representative	Largest tertiary hospital and training centre for medical postgraduate training.	Yes
Liberian College of Physicians and Surgeons: key representative	Institution involved in co-ordinating the medical postgraduate programme.	Yes
WHO: key representative	United Nations (UN) agency important for policy making, important donor to the medical sector.	Yes
United Nations Population Fund: key representative	UN agency important for policy making, important donor to the medical sector.	Yes
Nursing and Midwifery Board: key representative	Regulatory body of the nurses and midwives, including the COs.	Yes
Clinical obstetricians (COs)	Midwives trained in obstetric surgery.	Yes
Trainer COs	Gynaecologist training COs.	Yes
Hospital administrators	Non-medical lead within the hospital management.	Yes
National Public Health Institute of Liberia (NPHIL): key representative	In collaboration with the MOH, NPHIL strengthens existing infection prevention and control efforts, public health capacity building, response to outbreaks and monitoring of diseases with epidemic potential.	Yes
Ministry of Finance	Important for budget allocation towards the MOH.	No
Nurses	Another cadre that could be trained in surgery. Views assessed during the interview of the Nursing and Midwifery Board.	No
Law makers (Senate and House of Representatives)	Which could enact policy concerning surgical task sharing into practice.	No
Community	Final recipients of medical services.	No
Other donor organisations, like Partners in Health and MSF	NA.	No
US Agency for International Development: key representative	Important donor organisation to the medical sector of Liberia.	No

### Data analysis

The qualitative data was analysed using deductive coding and was coded using NVivo V.12. The deductive codebook was developed prior to analysing the qualitative data and was based on the themes as described within the semistructured questionnaire and using the constructs of the consolidated framework for implementation research (CFIR).^22^ The CFIR developed by Damschroder *et al*^23^ combines various implementation research frameworks together to assess an initiative, based within the context it operates. It assesses five domains: (1) the intervention characteristics; (2) the outer setting; (3) the inner setting; (4) characteristics of individuals; and (5) the process of implementation. It can be used during different phases of implementation: pre-implementation, mid-implementation and post implementation.^24^ For this study, the CFIR was used to group findings in one of its main domains using the CFIR guide.^22^ The process of implementation was not described as this is a pre-implementation study.

## Results

### Intervention characteristics

The core components are the essential elements of the intervention, and the adaptable periphery are the elements that could be changed.

Multiple participants considered surgery to be ‘an art’ and considered outcomes of surgeries to be the same for MOs and surgical specialists compared with PAs if trained in surgery. It was said that even some doctors were never officially trained in surgery. On the other hand, there were participants who expressed their reservations saying surgery is not only a mechanical thing of cutting but is also about understanding, for example, the physiology, preoperative management, resuscitation and the need for the surgical provider to be able to handle their own complications and that doctors and specialists are better trained for that.

It was also suggested that the programme should start as a pilot programme and the outcomes of the surgeries should be assessed in order to decide whether or not to continue with the programme. It would also be important to know how exactly the curriculum would look like and who the trainers or supervisors would be and to consider their qualifications. Most participants agreed that PAs trained in surgery should, in principal, strengthen the public sector, at least for the first years after graduation. Reservations towards working in private for-profit clinics were expressed as many times proper supervision would not be available in those clinics.

Overall, there was a preference to have surgically trained PAs work in rural underserved areas where there is a shortage of MOs. Additionally, there would be more surgical cases available because of a higher unmet surgical need. It was also thought that PAs, as it was their original mission, would be more willing to accept rural assignments as compared with MOs.

Several participants proposed the duration of a surgical training programme for PAs to be time bound, being a temporary solution. Durations between 3 and 30 years were proposed, until enough MOs would be trained.

The question was also raised that it would be depending on which procedure would be taught to PAs and depending on the evidence-based evaluation available from a similar programme in Sierra Leone if PAs would be able to deliver similar health outcomes compared with MOs.

Participants generally agreed that surgical training for PAs should prioritise life-saving or emergency procedures commonly encountered. Obstetrical emergencies such as caesarean sections, placenta removal and dilation and curettage were suggested, along with hernia surgery for general procedures. Regarding more specialised surgeries like laparotomies, hysterectomies, bowel resections and anastomosis, opinions varied on whether PA training should encompass these areas. Proponents argued for inclusion, particularly in rural settings, to mitigate referral delays in the absence of a national ambulance system and poor rural road conditions.

Multiple participants stated ideas about programme costs as an important adaptable element of the intervention, which are summarised in box 1.

Box 1 **The proposed areas to be budgeted within a surgical training programme for PAs, as suggested by various participants**

- Tuition fee of surgical training programme to be paid by participant or (partly) subsidised.
- Rehabilitation of training centre.
- Incentive for students (housing and living costs).
- Salaries of students and graduates.
- Salaries of trainers (local and expat).
- Training material.
- Building new surgical infrastructure like health centres in which graduates can work.
- Ensure supply of surgical and anaesthesia tools.
- Capacitating regulatory body.

### Outer setting

Outer setting includes the ‘economic, political and social context within which the implemented programme will interact’. Associated constructs include ‘peer pressure’ and external policies and incentives.

Surgical task-sharing initiative for midwifes (COs):

The existing COs surgical training programme for midwives was being criticised by various key stakeholders. Before the start of the COs’ programme in 2013, there was a stakeholders meeting in Bomi county; however, which stakeholders were exactly involved in the meeting is unknown. At that time, some stakeholders said ‘consensus’ to start the surgical task-sharing programme was reached.

The leadership of the LMDC argued, however, that the decision to start the CO programme was made on consensus by a few powerful stakeholders without the support of the MOs in general. This was one of the reasons why the LMDC refused to license the COs in the past. Therefore, the MOH in collaboration with Maternal and Child health Advocacy International decided to transfer the regulatory and licensing body of the COs towards the Nursing and Midwifery Board.

Further critique towards the COs’ programme varied from the opinion of the availability of a sufficient number of doctors to be able to do the obstetric surgeries, COs ‘taking’ the obstetric cases from intern doctors, COs being paid too much in relation to medical interns to the lack of institutionalisation (having a relation with a national university) of the COs’ programme and lacking a BSc degree for CO graduates.

It was suggested that the resistance against the COs’ programme was highly political, possibly because of the current plan of extending the programme of COs in the near future. The argument was made that the resistance was solely against the COs and not, for example, against the nurse anaesthetists or the nurses that are trained to perform cataract surgery. There were only few statements made about PAs already performing surgeries in the country.

They (MOs) trust a nurse to operate an eye, but you don’t trust somebody to make a big abdominal incision and take out a baby. You know, I mean I would rather give you the knife to do a cesarean section quickly than to give you the knife to work on my eye, you know, yeah but, so it is that kind of a paradox that we have. (Surgical specialist)

A former key stakeholder of the LMDC explained that prior to the start of the surgical task-sharing programme for COs, the concept of surgical task sharing in Liberia was explored by the MOH. Several visits took place by the key stakeholders and law makers of Liberia to African countries already implementing surgical task sharing, that is, Mozambique, Ethiopia and Zambia, but no act was passed into law.

### Inner setting

Inner setting includes the ‘structural, political and cultural context through which the intervention proceeds’ and the relationship between these elements. This involves the implementation climate and the individuals involved.

Multiple suggestions were made by stakeholders and their organisations to collaborate with a surgical training programme for PAs, for example, by the Liberian College of Physicians and Surgeons (LCPS) and PA training institutions. Both United Nations Family Planning Agency (UNFPA) and WHO as potential donors of the programme showed interest in the idea of training PAs in surgery. During the research, a first-level rural hospital was offered by its management to function as a training facility for the programme. All PAs interviewed were enthusiastic about the idea of starting a surgical training programme targeting their cadre as this could give them new career opportunities. By various stakeholders it was described that PAs were often not deployed, not paid or not paid on time. The unemployment rate of PAs was estimated to be between 30% and 40%, by the representative of a PA training institution and the representative of the LMDC. The high unemployment among PAs was one of the key reasons for stakeholders to support the idea of giving them additional skills in surgery as described below.

I think, for me it is okay […] in Liberia right now physician assistants do not have a career ladder, they are trained generally and after training they should really be assigned in rural health facilities where they can be there to support where medical doctors cannot reach. Right now, we even see after training it is also a challenge for government to employ them to go to those areas and we find out that because of this frustration many of them are turning into other professions. (Representative of a PA training institution)

The weak economic status of the country influences the *availability of resources*. A United Nations representative commented that it would be key for the government to buy-in (*leadership engagement),* to make the programme sustainable and to absorb the programme within the national budget. It was said that an economy that is weak would not deliver much revenue to government and would limit the capacity for government expenditure towards the programme. A representative of the MOH commented on its current financial challenges.

We have a limited budget. So, running a healthcare service is very difficult and I can give you a typical example; last year our total budget for the ministry of health was 63 million dollars, out of these 63 million dollars we only got 46 million dollars. Out of that 46 million dollars 39 million dollar went towards salary payment of health care workers. Running a whole health system was on seven million dollar that is not sustainable […]. How do you introduce more financial burden on the very weak financial system that the government has? (Representative MOH)

Recently, donors who previously were contributing towards paying salaries of healthcare workers in a pooled fund pulled out leaving a gap in the MOH’s budget.

### Characteristics of individuals

The individuals responsible for carrying out the intervention or otherwise related to the intervention, their agency and their relationships to each other and the intervention. Including knowledge and believes about surgical task sharing.

#### Medical doctors (MOs)

From all participants interviewed for this study, four were clearly against the concept of starting a surgical task-sharing programme for PAs in Liberia. All four opposing participants were MOs. The main argument put forward was that human resources are not the most pressing challenge of the surgical healthcare system, but surgical infrastructure is. Output of the medical school has been increasing steadily and specialists’ surgeons and gynaecologists are being trained. On the other hand, government was criticised for not paying the doctors sufficiently or on time, leaving the doctors unmotivated to take their (rural) assignments.

The main challenge stated by almost all participants was the resistance from the MOs to the start of a surgical task-sharing programme for PAs. The fear that patients and thereby salary would be taken away by a newly trained cadre was argued. A representative of the WHO described the factors leading to doctors’ resistance could be divided in two groups: one group of doctors being genuine willing to control the quality of the whole and the other group of doctors only willing to protect their own territory.

#### Ministry of Health

Multiple stakeholders within the MOH were interviewed. One important MOH representative was not in favour of training PAs in surgery. Again, poor surgical infrastructure was considered the main bottleneck of the surgical healthcare system, not shortage of human resources. Another challenge described was the weak financial resources of the government to absorb the programme within the government budget. It was mentioned that using a low doctor to population ratio as argument to train PAs in surgery would not be appropriate as many medical tasks are already shifted to nurses and PAs. Therefore, these cadres should be included in the doctor to population ratio.

## Discussion

**Figure 2:**
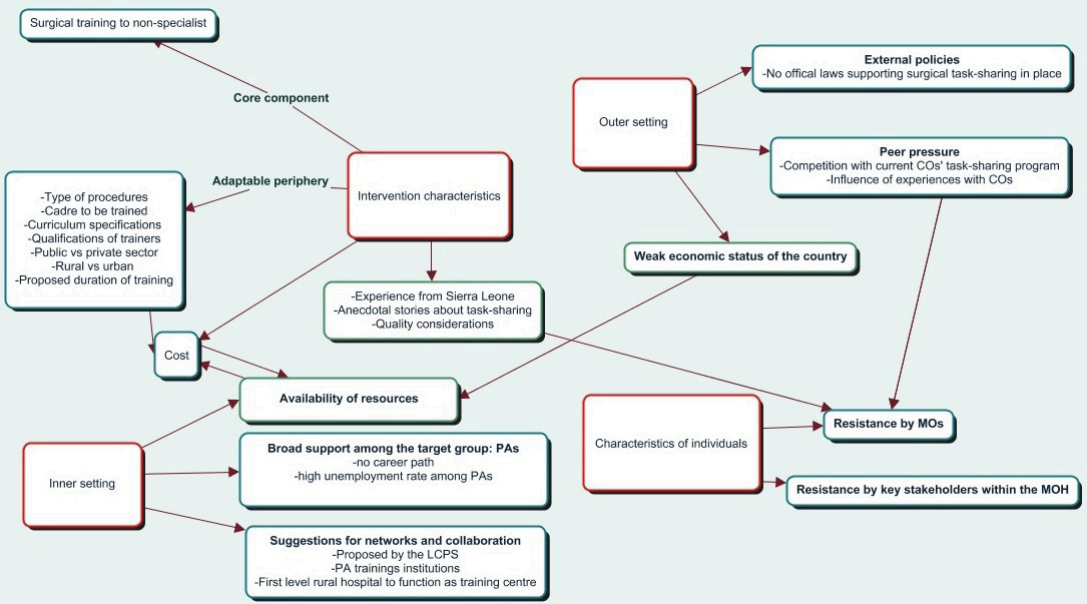
Simplified overview and relations of results grouped within the four domains of the consolidated framework for implementation research. COs, clinical obstetricians; MOs, medical officers; MOH, Ministry of Health; PAs, physician assistants; LCPS, Liberian College of Physicians and Surgeons.

In figure 2, the main results are grouped within the four domains of the CFIR. We are aware the domains could overlap and many more inter-relations do exist.

### Key enabling factors

#### Task sharing: not a novel approach

Surgical task sharing is not a novel concept within the healthcare system of Liberia, as both MOs and COs are already actively involved in performing surgeries. This pre-established practice has laid the groundwork for acceptance of the idea of training PAs in surgical procedures.

#### Career opportunities

Furthermore, the receptiveness of PAs to undergo surgical training is significant, as it offers them new career opportunities without significantly diverting them from their existing clinical duties, especially considering the substantial pool of unemployed PAs.

#### Proposed partnerships

The interest towards collaboration expressed by multiple stakeholders in the development of a surgical training programme for PAs is promising. Notably, partnerships with the postgraduate programme for physicians, as proposed by LCPS and John F. Kennedy Hospital, can help alleviate resistance among MOs. Integrating the programme into a PA training institution can contribute to its sustainability and institutionalisation reinforcing its long-term impact.

#### Increasing shortages of human resources for surgery

The WHO has pointed out that the shortages of healthcare professionals is expected to increase by 45% between 2013 and 2030 due to population growth in sub-Saharan Africa.^15^ Considering Liberia’s estimated population growth of approximately 3.3% year^25^ and the low number of graduates from both the medical school and the postgraduate training programmes, there is an urgent need to bolster the surgical workforce in the country. Based on international defined needs for surgeons, obstetricians and anaesthesiologists, Liberia would need about 900–1800 surgical providers in total. Even when all Liberian MOs are included as surgical providers, only 9%–18% of this target would be met.

#### Focus on the rural population

Prioritising the underprivileged and rural populations aligns with the sustainable development agenda of ‘Leaving no one behind’ and may lead to potential partnership with international organisations such as UNFPA and WHO.^26^ Such a focus on rural areas, the preference of most participants, could also increase support from doctors who may perceive less competition from the new cadre in urban areas where they are predominantly active.

### Key challenges

#### Negative experiences and leadership change

The turnover of leadership within the MOH, Liberian Medical and Dental Association and LMDC during the process of starting the existing surgical task-sharing programme for COs has given rise to ‘new’ resistance towards the concept of training mid-level clinicians in surgery. This highlights the importance of considering the opinions of new leaders and involving them in the programme’s development. As described by Saluja *et al*,^27^ Liberia’s top-down ministry engagement and the influential role of a few important individuals in decision-making processes underline the necessity of securing their support from the programme’s onset and maintaining their and associated organisations’ ongoing engagement. Continuous policy dialogues and evidence-based evaluations are critical for the long-term sustainability of such programmes. Support by only a few key figures is not enough.

#### Resistance by medical doctors (MOs)

The resistance towards the idea of training PAs in surgery is multifaceted. Ideas motivated by concerns of preserving professional territory were frequently posed.

Furthermore, MOs resistance to surgical task sharing is not unique to Liberia and has been observed in other West African regions as it is not as widely practised compared with other parts of Africa.^28^ Recommendations from experts advise that various healthcare provider groups (eg, representatives from the MOs) should be involved in the *design* of such interventions.^6^ Additionally, at the same time, enhancing the surgical training of MOs can prevent the shift of surgical cases from (not surgically trained) MOs to surgically trained associate clinicians, as witnessed in other countries.^11^

#### Surgical infrastructure

Some MOs and the MOH believe that the inadequacy of surgical infrastructure is a more immediate issue than the shortage of human resources for surgery. Surgical infrastructure encompasses the availability of resources like electricity, running water, hospitals, sterile tools and anaesthesia (including equipment). It can be assessed by a WHO Hospital Assessment Tool.^2^ However, evidence^13 18^ suggests that both human resources and infrastructure need to be strengthened in Liberia, emphasising the importance of a comprehensive approach for enhancing the surgical healthcare system.

### Political and economic considerations

The prevailing political and economic situation in Liberia poses significant challenges. During the time of the study major donors pulled out from a pooled fund to pay for healthcare worker salaries, inflation and strike actions have strained the country’s political and economic stability. In May 2019, donor funds were withdrawn and possibly not used as intended by the government,^29^ which may have contributed to the reluctance of donors to support the Liberian government. Additionally, during the field work of the study, there were signs that hospitals did not receive adequate supplies.^30^ The disproportionate allocation of government expenditure towards healthcare worker salaries and the subsequent non-payment of salaries have demotivated healthcare workers and made the government resistant to introducing a new cadre into the healthcare workforce. These economic and political factors underscore the complex environment in which efforts to enhance the surgical workforce must navigate.

### Study limitations

One of the limitations of this study was the limited comparability between the opinions of medical specialists. In Liberia, there are only a limited number of medical specialists available, which may hinder a comprehensive comparison of opinions. Two of the three gynaecologists that were interviewed for this study are involved in the training of COs, which might have resulted in a more supportive attitude towards surgical task-sharing compared with other specialists. Another limitation of this research is that patients and communities were not included in the interviews. Surgical task sharing is already common in Liberia, involving non-specialist physicians (MOs), midwives (COs) and anaesthetic nurses. Consequently, in general, it is recognised as an accepted intervention by patients and the community. Acceptance by the community is believed to be mainly dependent on quality of the service provided and emphasises the need for monitoring of the outcomes of any surgical task-sharing initiative.

Finally, the lead author (MJW) is a non-Liberian medical doctor with expertise in global health. Having a single researcher conduct the interviews might have introduced potential bias or subjectivity in the data collection process.

## Conclusion

Training of PAs in surgery is an opportunity to increase access to essential surgical services in Liberia. With PAs as the local champions advocating for a surgical training programme, their high unemployment rate and desire for career advancement could justify a surgical task-sharing programme targeting PAs. Additionally, various MOs, the Nursing and Midwifery Board, the LCPS, UNFPA and WHO were also in favour of starting a surgical training programme for PAs. Government support is fragile as there is no consensus within the MOH whether or not to support the idea of training PAs in surgery. Budgetary constraints and the opinion that the lack of surgical infrastructure is a more pressing problem were reasons for this division. Another challenge is the resistance from the MOs and their professional bodies. Factors for resistance are multiple and range from ‘genuine’ quality considerations to professional turf protection. Reservations from the MOs’ professional bodies with regard to the already implemented COs’ programme also have to be considered. If a new surgical training programme for PAs would be considered, it will be essential to align such initiative with the existing programme for COs. Further preparation of the intervention should eventually focus on adapting the ‘adaptable’ periphery in a way which broadens and strengthens the support of the MOH, MOs and their professional bodies towards the training of PAs in surgery. Failing to obtain such support should make the implementors consider alternative strategies to strengthen surgical human resources in rural Liberia.

## supplementary material

10.1136/bmjopen-2023-081363 online supplemental file 1

10.1136/bmjopen-2023-081363 online supplemental file 2

## Data Availability

The research data used in this study is available upon reasonable request and has been anonymised to protect the privacy and confidentiality of the participants. Please contact the corresponding author for inquiries regarding data access.
