# Supplementary material for: Exploring barriers and enabling factors for surgical task sharing with physician assistants in Liberia: a qualitative pre-implementation study
Source: BMJ Open. 2024 Jul 16;14(7):e081363. doi: 10.1136/bmjopen-2023-081363 (PMC11253748; doi:10.1136/bmjopen-2023-081363)
Supplement: online supplemental file 1 [file bmjopen-14-7-s001.pdf]

Type of training program	Entrance requirement	Duration of training	Recognition	Total duration	Output per year*
**A.M. Dogliotti medical college.**	BSc in natural or physical sciences (3-4 years)	5-years + a 2-years internship	Master degree	10-11 years	20-40 per year
**JFK hospital/ LCPS post-graduate training program.**	Medical officer	5 years	Specialist surgeon or gynecologist	15-16 years	2 surgeons and 2 gynecologists per year
**The Tubman National Institute of Medical Arts (TNIMA). Public PA training institute.**	Secondary school degree	3-years	Diploma degree	3-years	30 per year
**Baptist College of Missionary Physician Assistants (BSMPA). Public PA training institute.**	Secondary school	3-years	Diploma degree	3-years	10 per year
**The Cuttington University School of PAs (CUSPA). Private PA training institute.**	Secondary school	4-years	In transition from diploma to BSc degree	4-years	30 per year
**Training program for clinical obstetricians (COs) in Liberia.**	Midwives with at least a few years of experience	3-years	Diploma degree	Depending on experience prior to start of training (+- 8 years)	1-2 per year
**Surgical training program for associate clinicians in Sierra Leone. Supported by CapaCare.**	Associate clinician with at least 2-years of experience.	3-years	Diploma degree	8-years	5 per year

Supplementary material 1. Comparing specifications of relevant training programs in Liberia and Sierra Leone. *As an estimate reported by various interviewees over the year 2019.
