# Supplementary material for: Exploring barriers and enabling factors for surgical task sharing with physician assistants in Liberia: a qualitative pre-implementation study
Source: BMJ Open. 2024 Jul 16;14(7):e081363. doi: 10.1136/bmjopen-2023-081363 (PMC11253748; doi:10.1136/bmjopen-2023-081363)
Supplement: online supplemental file 2 [file bmjopen-14-7-s002.pdf]

Themes	Probes	Questions
Acceptability	Knowledge	• How difficult is it to have surgery, when needed in Liberia?<br>• What are the factors that make it difficult to have surgery in Liberia?<br>• What are the main challenges for the surgical health care system in Liberia?<br>• What do you think could be solutions to the human resource gap within the field of surgery in Liberia?<br>• Are you familiar with the concept of surgical task shifting? (if no: explain) if yes, see below.<br>• What do you understand from the concept of surgical task shifting?
	Experience	• What is your experience with surgical task-shifting?<br>• What do you think about surgical task shifting? And why? And what about training PAs in surgery?
	Attitude	• Would you support a surgical training program for PAs? If no, why not? If yes, see below.<br>• How would you support a surgical training program for PAs?
	Quality	• Do you think a thoroughly trained PA in surgery could deliver similar health outcomes compared to a medical doctor, why yes or no?<br>If need further clarification: under which circumstances?
	Competition	• How will the new surgical cadre create competition with other medical cadres? What could be solutions to this?
Feasibility	Challenges	• What could be challenges when starting a surgical training program for PAs?
	In relation to the educational system	• How do you think a curriculum for PAs trained in surgery should look like?<br>• What could be challenges in the development of a training curriculum?

		• How should supervision and continuous training of the newly trained cadre be organized?<br>• For medical doctors or specialists: how much responsibility would you give a well-functioning, surgically trained PA or surgically trained midwife?
	Recognition	• How should the new cadre be recognized? Why? (Bsc?)
	Regulation	• How should the new cadre be regulated?<br>• Will there be need for new legislation?
	Remuneration	• What should be the salary of a surgical trained PA in relation to PAs not trained and medical doctors? Who will have to pay for this? (Donor or government?)
	Referral system	• What possibilities are there of referring complicated surgical cases?
	Benefits	• What could be benefits of training PAs in surgical task shifting?
Appropriateness	General versus obstetric surgery (types of procedures)	• Which surgical procedures would be accepted to be taught to PAs in surgical training, if any at all? Why these operations?
	Midwifes versus Pas	• Some midwifes are already trained in surgery, how do you think another program for PAs should be combined with this program?
	Rural versus urban	• How does the need for surgical task-shifting differs between rural and urban?
	Public versus private	• What is your view on the new surgical cadre be working in the public sector?<br>• And what about the private sector?<br>• What could be pros and cons?
	Complementary necessary workforce	• Is there enough anesthetic workforce to support the newly trained surgical cadre? If no, what could be solution to this?

	Gender distribution in training program	• What do you think about the need for women to be trained as surgical PAs?
Costs/sustainability	Funding	• What are the necessary financial resources to start and continue a surgical training program for PAs?<br>• What could be challenges and opportunities for funding?<br>• Which organizations might be interested in collaboration?
	Sustainability	• In what form would you prefer surgical task shifting to exist in the far future, when possibly more doctors are trained?<br>• How to make a surgical task shifting program sustainable?
Power relations	Influential actors	• Who are the most influential actors in the field of surgical task shifting? And why?<br>• Which players/ stakeholders could facilitate a program focusing on training PA within the field of surgery?<br>• Which players/ stakeholders could oppose a program focusing on training PA within the field of surgery?<br>• How do these stakeholders interact with each other?<br>• What can be reasons not to support the surgical task shifting to PAs?<br>• What can be reasons to support the surgical task shifting to PAs?
Adoption		• How did the tendency of government to support or not support surgical task shifting develop from the past to where we are now and what should we expect for the future? Why?
Fidelity	Lessons to be learned from MCAI	• Is the current MCAI program different than originally set up? And how is it different?

Supplementary material 2. Semi-structered interview guide.
