# Supplementary material for: Exploring barriers and enabling factors for surgical task sharing with physician assistants in Liberia: a qualitative pre-implementation study
Source: BMJ Open. 2024 Jul 16;14(7):e081363. doi: 10.1136/bmjopen-2023-081363 (PMC11253748; doi:10.1136/bmjopen-2023-081363)
Supplement: Supplementary file 3 [file bmjopen-2023-081363.reviewer_comments.pdf]

PEER REVIEW HISTORY

BMJ Open publishes all reviews undertaken for accepted manuscripts. Reviewers are asked to complete a checklist review form (**http://bmjopen.bmj.com/site/about/resources/checklist.pdf**) and are provided with free text boxes to elaborate on their assessment. These free text comments are reproduced below.

This paper was submitted to a another journal from BMJ but declined for publication following peer review. The authors addressed the reviewers' comments and submitted the revised paper to BMJ Open. The paper was subsequently accepted for publication at BMJ Open.

ARTICLE DETAILS

**TITLE (PROVISIONAL)**	Exploring barriers and enabling factors for surgical task-sharing with Physician Assistants in Liberia: a qualitative pre-implementation study.
**AUTHORS**	Werz, Markus Jan; van Duinen, Alex; Hampaye, Theophilus; van den Broek, Ankie; Bolkan, Håkon

VERSION 1 – REVIEW

**REVIEWER**	Gajewski, Jakub<br>Royal College of Surgeons in Ireland
**REVIEW RETURNED**	05-Dec-2023

**GENERAL COMMENTS**	This is a very well written and interesing paper.<br><br>There are only minor comments:<br>spell in full INGO the first time it appears<br>check the language ege. p12 line should start with Continuous not continues.

**REVIEWER**	Bijlmakers, Leon<br>Radboud University Medical Center, Radboud Institute for Health Sciences (RIHS), Health Evidence
**REVIEW RETURNED**	15-Jan-2024

**GENERAL COMMENTS**	The topic of this paper is relevant, but there are several conceptual issues. First of all the title: it doesn't accurately reflect what the study actually covered. I would argue that the study explored 'perceptions' (or views, opinions) of a variety of stakeholders in Liberia about surgical task sharing – which are not necessarily identical to the actual barriers to and enablers of surgical task-sharing.<br><br>The paper is not sufficiently clear about the precise topic of interest, and I suspect that may have affected the interviews as well: was it the possible implementation of a surgical task-sharing training programme for PAs that interviewees were asked about (as the study aim at the end of the Intro section suggests); or was it – a little broader – the concept of surgical task-sharing itself?<br><br>There is a difference between (surgical) task sharing (WITH another professional cadre) and task shifting (TO another cadre),

	although that difference is not always made that clearly in the literature – which is unfortunate. ('Task-sharing to', as in the title of your paper, sounds odd, not proper English.) In task shifting, tasks are delegated or transferred, while in task sharing, tasks are delivered collaboratively by different staff categories. See for example Orkin et al.: Conceptual framework for task shifting and task sharing: An international Delphi study. Hum. Resour. Health 2021;19:61. In the context of Liberia, where rural health facilities do not have surgical specialists, I presume the term task shifting is more appropriate. In larger referral centres, task sharing may be practised, so as to lessen the burden of work on the 1 or 2 surgical specialists that are available. I wonder if there was any such nuance in your interview schedule. And, was such a distinction made by any of the respondents?<br><br>The paragraph on Surgical task-sharing in Liberia would need to be more specific about physician assistants (PA) and PA training provided by the three existing institutions: what does this training involve? PA's: is it a single cadre? Aren't there different types of PA, e.g. for clinical services, for obstetrical care, or perhaps PA that are trained for other tasks? What are their skills (if they practice in the public or private sector)? At what level of the health pyramid/system in Liberia are they deployed?<br><br>Also, as you are probably well aware, there are at least two distinct specialists involved in the provision of surgery in rural hospitals: general surgeons and obstetricians. Although you somehow do acknowledge that, I'm missing findings or at least a reflection (in the Discussion section) on similarities and differences, if any, how these two cadres view surgical task shifting (or sharing).<br><br>The Article summary (4th bullet point under Strengths and limitations) states that "surgical task sharing is already widely practiced in Liberia" (also mentioned in slightly different terms in the first sentence of the Discussion section, under Key enabling factors): these statements cry for evidence and references! The Intro section would need to at least mention that surgery performed by PAs is not an uncommon, or perhaps even a widespread phenomenon. How widespread is it? If there are no reliable data about surgical procedures performed by PAs, this at least needs to be mentioned. In addition, the Intro section (or the Study setting paragraph of the Methods section) would need to clarify whether MOs without (surgical) specialization ("with the majority of them practicing surgery") – or other PAs – receive any surgical training at all.<br><br>You are using the term 'Implementation research', but I'm not sure whether that is really applicable: what is it that is actually being implemented? Is it the (hypothetical) surgical training of PAs that would facilitate task-sharing/shifting? Or is it the entire concept of surgical task-sharing See also my earlier comment.<br><br>The Results section appears to go much beyond a presentation of the findings of the key informant interviews. Table 1, for example, contains analyses performed by the researchers after completion of the interviews. Hence it rather belongs to the Discussion section. Had the researchers subjected the relations shown in Figure 2 to a discussion or individual feedback from (some of) the key informants, it might have enriched the study. And you would probably have come to realise that some of the relations are

	bidirectional rather than simply unidirectional; and that there may also be circular loops. See for instance the work done by SURG-Africa; e.g. three papers on group model building around the provision of surgery in LMIC settings, all led by Broekhuizen, incl one in Tanzania (doi: 10.1371/journal.pone.0257597), one in Malawi (doi: 10.34172/ijhpm.2021.78) and another one in Zambia (doi 10.1371/journal.pone.0257597).<br><br>The Key enabling factors and Key challenges in the Discussion section are a bit hard to identify, in particular the challenges: there seems to be overlap between the three (two?) types of resistance that are described.<br><br>The first sentence of the Conclusion ("The overall consensus is that surgical access has to be increased in Liberia."), although probably correct, is not something one would expect in view of the study aim. Last sentence: should 'the implementors' consider alternative strategies (implementors of what?) or is this recommendation rather for (health) policymakers?<br><br>Language: several issues. 'Resistors' is not a term that is commonly used in scientific literature. Is it any different from 'barriers' (in the title)?

**REVIEWER**	Wagstaff, Duncan<br>University College London Research Department of Epidemiology and Public Health
**REVIEW RETURNED**	14-Feb-2024

**GENERAL COMMENTS**	This is a valuable and timely contribution to an important subject. It is well-designed and well-executed. I and have only minor amendments to suggest.<br><br>Suggestions:<br>1. I would prefer a dedicated paragraph to discuss the limitations outlined in the article summary.<br>2. 'Surgical infrastructure' is repeatedly referred to as a separate/competing challenge to workforce constraints, but it is not defined – please could it be defined<br>3. P3 line22 'it is already known to be an accepted intervention by patients and the community' – please provide evidence in the manuscript to support this claim<br>4. It would be informative to have more detail about the context, implementation and impacts of surgical task-sharing to PAs in Sierra Leone<br>5. Results 'intervention characteristics': it would be useful to know how the factors described here have played out during the implementation of the clinical obstetricians<br><br><br>Typos – multiple, including:<br>P1 line 57 ' believes' should be 'beliefs'<br>P3 line 3 'decide' should be 'deciding', and delete 'a'<br>P6 Line 28 – 'trainings' should be 'training'<br>P6 line 30 – change full stop to comma before suggesting<br>P6 line 30 – was/were<br>P6 line 37 – add 'the' before Liberian

**REVIEWER**	STAVERT, BETHANY

	The University of Sydney, Health and Medical Sciences
**REVIEW RETURNED**	19-Feb-2024

**GENERAL COMMENTS**	1. Methodology- it was unclear why the decision for the methodology was made. It was not explained and the headings using the methodology did not link well with what was covered. This needs to be explored- so that the reader can understand the lens and flow of ideas better.<br>2. Methods: It would be helpful to have the interview questionaire attached so the reader can see what was asked during the interview, to explain further the results.<br>3. Limitations: BIAS- there was some bias in this paper but this was not fully discussed. The one researcher was medical and not surgical. Whilst the PA need for jobs was discussed multiple times and referenced, the conditions for doctors, how many were graduating, the infrastructure needed to employ further PAs... why would a PA get paid when doctors are not getting paid?<br>Also this is an extension of an obstetrics initiative- which already has a strong midwife culture. The limitations/differences between surgery and obstetrics should be noted.<br>4.To note there are some grammatical errors, minor changes should be made (line 42 page 9, line 3 page 3, line 37 page 6).<br>5. What the surgical task-sharing would look like. It is unclear what task sharing means- is this working under the supervision of a surgeon or independently? Is it for basic surgery that is referred by surgeon or is it stand alone separate practice.

VERSION 1 – AUTHOR RESPONSE

Reviewer: 1
Dr. Jakub Gajewski, Royal College of Surgeons in Ireland
Comments to the Author:
This is a very well written and interesting paper.

1. There are only minor comments:
spell in full INGO the first time it appears
check the language ege. p12 line should start with Continuous not continues.

The proposed corrections have been incorporated into the original document

Reviewer: 2
Dr. Leon Bijlmakers, Radboud University Medical Center, Radboud Institute for Health Sciences (RIHS)
Comments to the Author:
The topic of this paper is relevant, but there are several conceptual issues.

1. First of all the title: it doesn’t accurately reflect what the study actually covered. I would argue that the study explored ‘perceptions’ (or views, opinions) of a variety of stakeholders in Liberia about surgical task sharing – which are not necessarily identical to the actual barriers to and enablers of surgical task-sharing.

The titles was changed to:
Exploring barriers and enabling factors for surgical task-sharing with Physician Assistants in Liberia: a qualitative pre-implementation study.

2. The paper is not sufficiently clear about the precise topic of interest, and I suspect that may have affected the interviews as well: was it the possible implementation of a surgical task-sharing training programme for PAs that interviewees were asked about (as the study aim at the end of the Intro section suggests); or was it – a little broader – the concept of surgical task-sharing itself?

The original research was part of a master's thesis in 'International Health,' with the title of the thesis being: 'What are the views among the key stakeholders on surgical task-shifting in Liberia?' Due to the substantial amount of data generated—too much for a single article—and the study's collaboration with CapaCare (see further below and additional information incorporated in manuscript), who were eager to explore the idea of initiating a surgical training program for physician assistants in Liberia, I primarily focused on this aspect. Therefore, I adjusted the title of the article and analyzed the described outcomes accordingly, using the CFIR framework. For better understanding, I have included the study questionnaire as an addendum.

3. There is a difference between (surgical) task sharing (WITH another professional cadre) and task shifting (TO another cadre), although that difference is not always made that clearly in the literature – which is unfortunate. (‘Task-sharing to’, as in the title of your paper, sounds odd, not proper English.) In task shifting, tasks are delegated or transferred, while in task sharing, tasks are delivered collaboratively by different staff categories. See for example Orkin et al.: Conceptual framework for task shifting and task sharing: An international Delphi study. Hum. Resour. Health 2021;19:61. In the context of Liberia, where rural health facilities do not have surgical specialists, I presume the term task shifting is more appropriate. In larger referral centres, task sharing may be practised, so as to lessen the burden of work on the 1 or 2 surgical specialists that are available. I wonder if there was any such nuance in your interview schedule. And, was such a distinction made by any of the respondents?

Internally, we also debated which term to use: 'task sharing' or 'task shifting'. You are correct that in rural Liberian first level hospitals, there are no specialist, however there are non-specialist MDs performing surgery. Our thinking was that PAs shared surgical tasks with them. Thus, we settled on the term 'task sharing', also given the fact that team-work will be a critical element in effective collaboration between cadres (Is task sharing preferred to task shifting in the provision of safe surgical care? Smith. Surgery. 2018 Sep;164(3):559-560. doi: 10.1016/j.surg.2018.05.005

We have changed the title to ‘
Exploring barriers and enabling factors for surgical task-sharing with Physician Assistants in Liberia: a qualitative pre-implementation study.

Furthermore, we have added in the method section the sentence: In the literature, the terms ‘task-shifting’ and ‘task-sharing’ are used interchangeably. In this manuscript we consciously choose the term ‘task-sharing’ as this underlines a broader systemic approach and the necessary support from medical officers to deliver safe and high-quality surgical care by a newly trained cadre (18).

Additionally, the article of Orkin et al. you refer to states that: ‘Another eight (of the 16) panelists indicated that task shifting and task sharing refer to the same phenomenon, but that task sharing is the preferred term because it captures the intrinsically collaborative nature of these undertakings.’ We agree to this statement.

4. The paragraph on Surgical task-sharing in Liberia would need to be more specific about physician assistants (PA) and PA training provided by the three existing institutions: what does this training involve? PA’s: is it a single cadre? Aren’t there different types of PA, e.g. for clinical services, for

obstetrical care, or perhaps PA that are trained for other tasks? What are their skills (if they practice in the public or private sector)? At what level of the health pyramid/system in Liberia are they deployed?

We have adjusted and extended the description of the PAs training programs and added a table with more details. An overarching PA association oversees that the necessary subjects with regard to clinical medicine and primary health care are covered by the schools' curricula. Up till now the curricula are not the same for the different schools. For example, to be able to offer a BSc degree the school will need collaboration with a university and some standard subjects like English language and mathematics to be taught. A secondary school diploma is necessary for entrance.

The following paragraph and supplementary material 1 have been added:
Since 1958 Liberia has implemented a program for PAs. PAs work mostly independently, especially, at (rural)health posts, clinics and health centers using the basic concepts of primary health care. In places where no trained midwife is available he or she could also provide basic obstetrical care. At the moment Liberia has 3 PA trainings institutions (see table 1). In 2019 there were 1036 registered PAs, of which 532 were actively practicing clinical medicine. Suggesting many PAs are not practicing or not being captured as practicing (49%). Of the group practicing 75% is working in the public and 25% in the private sector. From the group working in the public sector 80% is working in primary health care and the other 20% in the hospitals (source: PA association). The exact number of PAs involved in surgery or independently performing surgeries is unknown but expected to be low.

5. Also, as you are probably well aware, there are at least two distinct specialists involved in the provision of surgery in rural hospitals: general surgeons and obstetricians. Although you somehow do acknowledge that, I'm missing findings or at least a reflection (in the Discussion section) on similarities and differences, if any, how these two cadres view surgical task shifting (or sharing).

Thank you for bringing up this point.
I refer to my answer to your next question (comment 6).
Additionally, see comment 1 of reviewer 3. Added paragraph of study limitation to the discussion.

6. The Article summary (4th bullet point under Strengths and limitations) states that "surgical task sharing is already widely practiced in Liberia" (also mentioned in slightly different terms in the first sentence of the Discussion section, under Key enabling factors): these statements cry for evidence and references! The Intro section would need to at least mention that surgery performed by PAs is not an uncommon, or perhaps even a widespread phenomenon. How widespread is it? If there are no reliable data about surgical procedures performed by PAs, this at least needs to be mentioned. In addition, the Intro section (or the Study setting paragraph of the Methods section) would need to clarify whether MOs without (surgical) specialization ("with the majority of them practicing surgery") – or other PAs – receive any surgical training at all.

Thank for this feedback, we were clearly not able to explain this in the first submitted manuscript. With task sharing being practiced widely we didn't mean practiced by PAs. During the interviews there were only few statements made about PAs already performing surgeries in the country.

The statement (4th bullet point under strengths and limitations) will be changed from:
[..] As surgical task sharing is already widely practiced in Liberia it is already known to be an accepted intervention by patients and the community.
To: Surgical task sharing is already common in Liberia, involving non-specialist physicians, midwives, and anesthetic nurses. Consequently, in general it is recognized as an accepted intervention by patients and the community.

Surgical task sharing is already extensively practiced in Liberia. The majority of surgeries (58.3%) are performed by non-specialist physicians instead of medical specialists. Additionally, at the time of the interviews there was a task-sharing initiative where midwives were trained in surgical obstetric procedures. This program is now closed. Interviews revealed that physician assistants also occasionally perform surgical procedures, although these are rare occurrences.
Anesthesia is performed by anesthetic nurses trained at Phebe hospital (see: https://www.phebeschool.org/menu_info.php?&02467bd826cafd89e1a2700e5874f0ec=MjQ%3D).

For reference see:
Adde HA et al. A Nationwide Enumeration of the Surgical Workforce, its Production and Disparities in Operative Productivity in Liberia. World J Surg. 2022.

A part of the method section study setting has been removed:
"In 2017 there were 298 registered MOs in Liberia, of those, 12 were surgeons and 10 were gynecologists. The country lacks specialist anesthesiologists and anesthesia is given by anesthetic nurses (source: Liberian Medical and Dental Council). In Liberia, surgical tasks are performed by MOs without specialization, with the majority of them practicing surgery."

And was replaced by and moved under the subheading surgical task sharing:
In 2018, Liberia had 286 registered surgical providers, including 67 medical specialists and 19 non-physicians. Including three anesthesiologist specialists. Areas with higher poverty had fewer specialists (0.7 per 100,000) compared to less impoverished areas (3.6 per 100,000). Non-specialist physicians (MOs) performed 58.3% of surgeries (16). A 6 months period during the training of MOs is dedicated to obtaining skills in emergency obstetrical surgeries and neonatal care.
Additionally, anesthesia is provided by anesthetic nurses trained at Phebe hospital.

Under the section Outer setting I will add some statements that suggest a lot of critique against 'surgical task sharing' is thought to be political and mainly focused against the surgical training program for clinical obstetricians.

It was suggested that the resistance against the COs' program was highly political. Possibly because of the current plan of extending the program of COs in the near future. The argument was made that the resistance was solely against the COs and not for example against the nurse anesthetists or the nurses that are trained to perform cataract surgery.
"They (MOs) trust a nurse to operate an eye, but you don't trust somebody to make a big abdominal incision and take out a baby. You know, I mean I would rather give you the knife to do a cesarean section quickly to give you the knife to work on my eye, you know, yeah but, so it is that kind of a paradox that we have." (Surgical specialist).

7. You are using the term 'Implementation research', but I'm not sure whether that is really applicable: what is it that is actually being implemented? Is it the (hypothetical) surgical training of PAs that would facilitate task-sharing/shifting? Or is it the entire concept of surgical task-sharing See also my earlier comment.

We have called this a "a qualitative, pre-implementation study using semi-structured interviews". I would like to refer to: Means et al. Evaluating and optimizing the consolidated framework for implementation research (CFIR) for use in low- and middle-income countries: A systematic review. Implement Sci. 2020;15(1):1–19. Please see particularly Table 1, which cites multiple studies utilizing the CFIR in the pre-implementation stage, either for designing an intervention or assisting with data collection. For instance, to identify potential barriers and facilitators to implementing system changes to increase adoption of tobacco use treatment guidelines.

Furthermore, the findings of our current study have been utilized by CapaCare, an INGO already active in training associate clinicians in surgery in Sierra Leone. They wanted to explore the possibility of training Physician Assistants in surgery in Liberia. However, based on the outcomes, this initiative has been put on hold for now. Currently, they are focusing on training resident surgeons in Liberia through the Liberian College for Physicians and Surgeons. Perhaps, with broader acceptance among physicians and once CapaCare has established a reputation, the program may still be implemented in the future. Please also refer to CapaCare's annual report: https://capacare.org/wp-content/uploads/2016/12/CapaCare-Annual-Report-2022-low-res.pdf

We will add a sentence at the end of the introduction clarifying Capacare to be interested in exploring to extend its activities to the Liberian context:

Capacare an organization involved in training associate clinicians in surgery in Sierra Leone (14) was interested to explore the opportunity of extending its activities to the context of neighboring Liberia. Therefore, outcomes of this study were used to guide their strategic direction.

Adding a reference describing the Sierra Leone program its implementation. See:
Bolkan HA, van Duinen A, Waalewijn B, Elhassein M, Kamara TB, Deen GF, et al. Safety, productivity and predicted contribution of a surgical task-sharing programme in Sierra Leone. Br J Surg. 2017;104(10):1315–26.

8. The Results section appears to go much beyond a presentation of the findings of the key informant interviews. Table 1, for example, contains analyses performed by the researchers after completion of the interviews. Hence it rather belongs to the Discussion section. Had the researchers subjected the relations shown in Figure 2 to a discussion or individual feedback from (some of) the key informants, it might have enriched the study. And you would probably have come to realise that some of the relations are bidirectional rather than simply unidirectional; and that there may also be circular loops. See for instance the work done by SURG-Africa; e.g. three papers on group model building around the provision of surgery in LMIC settings, all led by Broekhuizen, incl one in Tanzania (doi: 10.1371/journal.pone.0257597), one in Malawi (doi: 10.34172/ijhpm.2021.78) and another one in Zambia (doi 10.1371/journal.pone.0257597).

It is true Table 1 which is in the method section contains additionally identified stakeholders through snowball sampling. Some of the interviews were performed after identification by this process. Therefore, we think it is still adequate to keep Table 1 under the method section.

Thank you for the interesting references to the work of Group Model Building (a form of focus group discussions) and surgical team mentoring.
We are aware of the complexity of the interactions within the surgical health care system. We prefer not to dive to deeply in feedback loops and bidirectional interactions as we don’t want to make the depicted findings too complex to interpret.

In my opinion figure 2 is an analysis of the findings in the different groups mentioned by the CFIR guide. Off course it is very subjective and grouped by a single researcher and as mentioned there is a lot of overlap between the different constructs.

We will place figure 2 under the section: discussion.

9. The Key enabling factors and Key challenges in the Discussion section are a bit hard to identify, in particular the challenges: there seems to be overlap between the three (two?) types of resistance that are described.

Sub headings have been included in the discussion section.

Key enabling factors:
Task-sharing; not a novel approach
Career opportunities
Proposed partnerships
Increasing shortage of human resources for surgery
Focus on the rural population

Key challenges:
Negative experiences and leadership change
Resistance by medical doctors
Surgical infrastructure

10. The first sentence of the Conclusion ("The overall consensus is that surgical access has to be increased in Liberia."), although probably correct, is not something one would expect in view of the study aim. Last sentence: should 'the implementors' consider alternative strategies (implementors of what?) or is this recommendation rather for (health) policymakers?

Thank you for this point, we have changed:
"The overall consensus is that surgical access has to be increased in Liberia" in;
Training of PAs in surgery is an opportunity to increase access to essential surgical services in Liberia.

See last edited paragraph of introduction and edited method section on Sierra Leone context.

11. Language: several issues. 'Resistors' is not a term that is commonly used in scientific literature. Is it any different from 'barriers' (in the title)?

We will change the word resistors into opponents (as used in the abstract of the manuscript).

Reviewer: 3
Dr. Duncan Wagstaff, University College London Research Department of Epidemiology and Public Health, National Institute of Academic Anaesthesia
Comments to the Author:
This is a valuable and timely contribution to an important subject. It is well-designed and well-executed. I and have only minor amendments to suggest.

Suggestions:
1. I would prefer a dedicated paragraph to discuss the limitations outlined in the article summary.

A paragraph was added under the discussion section:

One of the main limitations in this study was the limited comparability between the opinions of specialists from different specialties. In Liberia, there is only a limited number of medical specialists available, which may hinder a comprehensive comparison of opinions. Two of the three gynecologists that were interviewed for this study are involved in the training of COs, which might have resulted in a more supportive attitude towards surgical task-sharing compared to other specialists.

Another limitation of this research is that patients and communities were not included in the interviews. Surgical task sharing is already common in Liberia, involving non-specialist physicians, midwives, and anesthetic nurses. Consequently, in general it is recognized as an accepted

intervention by patients and the community. Acceptance by the community is mainly dependent on quality of the service provided and emphasizes the need for monitoring of the outcomes of any surgical task sharing initiative.

Finally, the primary author is non-Liberian medical doctor with expertise in global health. Having a single researcher conduct the interviews might have introduced potential bias or subjectivity in the data collection process.

2. ‘Surgical infrastructure’ is repeatedly referred to as a separate/competing challenge to workforce constraints, but it is not defined – please could it be defined

See edited paragraph of the discussion on surgical infrastructure.

Surgical infrastructure encompasses the availability of resources like electricity, running water, hospitals, sterile tools and anesthesia (including equipment). It can be assessed by a WHO Hospital Assessment Tool(2).

3. P3 line22 ‘it is already known to be an accepted intervention by patients and the community’ – please provide evidence in the manuscript to support this claim

See answer to comment 6, reviewer 2, Dr. Leon Bijlmakers, Radboud University Medical Center, Radboud Institute for Health Sciences and additional comments in added study limitations.

4. It would be informative to have more detail about the context, implementation and impacts of surgical task-sharing to PAs in Sierra Leone

See answer to comment 7, reviewer 2, Dr. Leon Bijlmakers, Radboud University Medical Center, Radboud Institute for Health Sciences and additional paragraph about the Sierra Leone context in the method section.

Sierra Leonean context
Sierra Leone and Liberia share similar healthcare challenges, including a high unmet need for surgery (20)(13) and weak healthcare systems. CapaCare, operating as both an international and national NGO, established in Sierra Leone in 2011 a surgical training program for associate clinicians in obstetric and general essential surgery. At the start, the training program was designed for Community Health Officers, a cadre comparable to PAs in Liberia, both working mainly in primary healthcare facilities. This program involves 12 months of basic training in a main training facility followed by clinical rotations in partner hospitals. After completing rotations and examinations, graduates undergo a one-year housemanship stage, split between tertiary hospitals in Freetown and district hospitals.

5. Results ‘intervention characteristics’: it would be useful to know how the factors described here have played out during the implementation of the clinical obstetricians

In the method section under task-sharing an additional phrase is added with reference to an article describing the implementation of the clinical obstetricians.
A description of the implementation of the program is described by Dolo et al(20). Within the training program the trainees started assisting senior doctors but progressed to independently manage obstetric surgeries.

See also the added table 1 for additional details.

Typos – multiple, including:

Corrected as proposed.

P1 line 57 ‘ believes’ should be ‘beliefs’
P3 line 3 ‘decide’ should be ‘deciding’, and delete ‘a’.
P6 Line 28 – ‘trainings’ should be ‘training’
P6 line 30 – change full stop to comma before suggesting
P6 line 30 – was/were
P6 line 37 – add ‘the’ before Liberian

Reviewer: 4
Dr. BETHANY STAVERT, The University of Sydney, Concord Repatriation General Hospital
Comments to the Author:
1. Methodology- it was unclear why the decision for the methodology was made. It was not explained and the headings using the methodology did not link well with what was covered. This needs to be explored- so that the reader can understand the lens and flow of ideas better.

See comment 4 of interviewer 2. Adding more background information on the study setting.
See comment 7 of interviewer 2. Linking the introduction to the methodology.
See comment 4 of interviewer 3. Adding more background information on the study setting.

Furthermore, a sentence was added under study design and data collection.

It explores the participants views on surgical task-sharing with special focus on the idea of implementing a ‘hypothetical’ surgical task-sharing training program for PAs in the future.

2. Methods: It would be helpful to have the interview questionaire attached so the reader can see what was asked during the interview, to explain further the results.

Added as a supplementary material 2.

3. Limitations: BIAS- there was some bias in this paper but this was not fully discussed. The one researcher was medical and not surgical. Whilst the PA need for jobs was discussed multiple times and referenced, the conditions for doctors, how many were graduating, the infrastructure needed to employ further PAs... why would a PA get paid when doctors are not getting paid?

See added supplementary material 1 for more details about the different training programs. I totally agree with the point that it will be difficult to pay a newly trained cadre if even the doctors cannot be paid. More money needs to come available to pay for salaries of health care workers. This has been addressed under political and economic considerations in the discussion section.

Also this is an extension of an obstetrics initiative- which already has a strong midwife culture. The limitations/differences between surgery and obstetrics should be noted.

This study has been set up to aid developing the intervention, therefore it is called a pre-implantation study. Within the Sierra Leonean setting, CapaCare trains associate clinicians in both general and obstetric surgeries. In Sierra Leone there is not a surgical training program for midwives. Therefore, as written in my conclusion of the manuscript it will be essential, when developing the intervention for the Liberian context a hypothetical surgical training program for PAs needs to be aligned with the surgical training program for midwives (clinical obstetricians).

The results section, intervention characteristics will be changed (red deleted and replaced by yellow). For the type of surgeries participants agreed that the training of PAs in surgery should focus on lifesaving or emergency procedures most frequently encountered. For more specialized procedures there was a division whether or not it would be appropriate to support training PAs into these areas. Participants generally agreed that surgical training for PAs should prioritize life-saving or emergency procedures commonly encountered. Obstetrical emergencies such as cesarean sections, placenta removal, and D&C were suggested, along with hernia surgery for general procedures. Regarding more specialized surgeries like laparotomies, hysterectomies, bowel resections, and anastomosis, opinions varied on whether PA training should encompass these areas. Proponents argued for inclusion, particularly in rural settings, to mitigate referral delays in the absence of a national ambulance system and poor rural road conditions.

4.To note there are some grammatical errors, minor changes should be made

Corrected as proposed
Line 42 page 9, mistake not found.
line 3 page 3,
line 37 page 6).

5. What the surgical task-sharing would look like. It is unclear what task sharing means- is this working under the supervision of a surgeon or independently? Is it for basic surgery that is referred by surgeon or is it stand alone separate practice.

See comment 3 of interviewer 1.

Furthermore, the question of the level of supervision was extensively addressed in the interviews: 'How should supervision and continuous training of the newly trained cadre be organized?'. It is true the country lacks medical specialist and most surgeries are performed by medical officers without specialization. This could be seen as a form of task shifting or task sharing, depending the term that is preferred.

Reviewer: 1
Competing interests of Reviewer: NONE

Reviewer: 2
Competing interests of Reviewer: Ì have no competing interests

Reviewer: 3
Competing interests of Reviewer: nil

Reviewer: 4
Competing interests of Reviewer: No competing interests, but it should be noted that I am a surgical trainee and may have some bias

Additional changes to the original submitted format:
Changed figure 1. Amount of gynecologists interviewed from 5 to 3 of which 2 were (former) trainers of clinical obstetricians.

We have changed the summary box; strengths and limitations as the editors suggested.

Editors comment: The novelty, aims, results or expected impact of the study should not be summarized here.

To:
Strengths and limitations
• The study addresses a critical issue in Liberia, where there is a severe shortage of surgical providers and surgical infrastructure, resulting in a low surgical volume.
• It explores the potential for surgical task-sharing to PAs to expand surgical workforce, which is a highly relevant and important topic in global health.
• The study explores the views of key stakeholders in the field of surgery or PAs and their training institutions on surgical task-sharing with special focus on the idea of implementing a surgical training program for PAs in the future.
• Utilization of the Consolidated Framework for Implementation Research (CFIR) adds a structured and theoretically informed approach to the study. It enhances the rigor of the research by providing a framework for organizing and analyzing the data.
• Surgical task sharing is already common in Liberia, involving non-specialist physicians, midwives, and anesthetic nurses. Consequently, in general it is recognized as an accepted intervention by patients and the community.
• The study acknowledges that patients and the community are important stakeholders as well but were not included in the interviews.
• While the lead author is an experienced medical doctor with expertise in global health and surgery, having a single researcher conduct the interviews might have introduced potential bias or subjectivity in the data collection process.

VERSION 2 – REVIEW

**REVIEWER**	Wagstaff, Duncan<br>University College London Research Department of Epidemiology and Public Health
**REVIEW RETURNED**	14-May-2024

**GENERAL COMMENTS**	Many thanks for the authors for adding clarity to the manuscript.<br><br>I have only minor comments/queries, mostly linguistic:<br>P3 ‘prior to develop’ should be ‘prior to developing’<br>P5 ‘trainings’ should be ‘training’<br>P5 ‘Including three anesthesiologist specialists’. This is not a sentence.<br>P6 ‘task-sharing to’ …should this be ‘task-sharing with’<br>P10: ‘Several participants proposed the duration of a surgical training program for PAs to be time bound, being a temporal solution’ …this doesn’t make sense. Do you mean ‘temporary’ instead of ‘temporal’?<br><br>I feel another good edit of the manuscript would improve grammar and syntax.

VERSION 2 – AUTHOR RESPONSE

Reviewer: 3
Dr. Duncan Wagstaff, University College London Research Department of Epidemiology and Public Health, National Institute of Academic Anaesthesia
Comments to the Author:
Many thanks for the authors for adding clarity to the manuscript.

1. I have only minor comments/queries, mostly linguistic:

Corrected as proposed.

P3 ‘prior to develop’ should be ‘prior to developing’
P5 ‘trainings’ should be ‘training’
P5 ‘Including three anesthesiologist specialists’. This is not a sentence.Removed from the manuscript.
P6 ‘task-sharing to’ …should this be ‘task-sharing with’
P10: ‘Several participants proposed the duration of a surgical training program for PAs to be time bound, being a temporal solution’ …this doesn’t make sense. Do you mean ‘temporary’ instead of ‘temporal’? Correct, thank you!

Additionally trainings institutions was changed to training institutions in table 1.

I feel another good edit of the manuscript would improve grammar and syntax.
Another check of grammar and syntax has been done (green in marked version).

Reviewer: 3
Competing interests of Reviewer: nil

Editor(s)' Comments to Author (if any):
1. Please revise the second point of your ‘Strengths and limitations of this study’ section of your manuscript (after the abstract). This section should contain up to five short bullet points, no longer than one sentence each, that relate specifically to the methods. The novelty, aims, results or expected impact of the study should not be summarised here.

Formatting Amendments (where applicable):

We revised the second point from:
• Surgical task sharing is already common in Liberia, involving non-specialist physicians, midwives, and anesthetic nurses. Consequently, in general it is recognized as an accepted intervention by patients and the community.
To:
• Authors' extensive experience with a task-sharing program in neighboring Sierra Leone enhances the credibility and depth of the study.
